# Psychological distress in clinical research professionals in acute care settings: Potential risks and future directions

**DOI:** 10.1017/cts.2025.10163

**Published:** 2025-09-25

**Authors:** Ian H. Stanley, Corey B. Bills, Adit A. Ginde

**Affiliations:** 1 Department of Emergency Medicine, University of Colorado Anschutz Medical Campus, Aurora, CO, USA; 2 Center for Combat Medicine and Battlefield (COMBAT) Research, University of Colorado Anschutz Medical Campus, Aurora, CO, USA

Clinical research in acute care settings, such as emergency departments (EDs), intensive care units (ICUs), and operating rooms, is vital to advance life-saving care for critically ill and injured patients. As part of this work, clinical research professionals (CRPs) – spanning study coordinators, research assistants, data managers, biostatisticians, regulatory specialists, and clinicians – may be confronted with distressing stimuli, including exposure to serious injuries or procedures, gruesome images, or unanticipated death, that increase their risk for serious psychological distress. Unmitigated psychological distress among research team members has myriad potential consequences for the individuals as well as the study. Psychological distress among CRPs can impact team cohesion, thereby impacting study operations, including patient enrollment and participation, and putting the study deliverables and timelines at risk. Yet, there has been limited attention to the psychological toll that conducting clinical research in acute care settings may exact on research personnel, specifically (whereas attention has been paid to the psychological risks for research participants [1]). In this viewpoint, we highlight a range of potential exposures that may increase the risk of various psychological outcomes among research teams, discuss potential psychological consequences, and propose a plan for the field.

## Exposure pathways

CRPs perform various duties: they consent patients, conduct baseline and follow-up assessments, administer study interventions, and analyze data, which may include images. The degree to which CRPs are exposed to situations that might be psychologically distressing is determined by various factors – the focus of the study’s aims, the patient populations involved, the recruitment settings, and one’s lived experiences. Studies in domains such as severe trauma, burn treatment, cardiac arrest, and respiratory failure frequently require CRPs to engage directly or indirectly with content that may be highly distressing and traumatic, which may be amplified in research related to military combat, where injuries can be severe.

CRPs may encounter these potentially distressing stimuli through various means. This could include team members’ direct contact with a participant’s injuries or illness, either at the enrollment, assessment, intervention, or follow-up stages of the research; repeatedly viewing images that are captured for data processing purposes (e.g., pictures of wounds to monitor infection risk and progression); or learning about the sudden death of a participant, especially proximal to a recent study visit.

## Potential psychological consequences

There is a lack of data regarding the psychological effects of clinical research on CRPs working in acute care settings. Many staff will either not experience significant distress or will experience mild, transient distress reactions that resolve without intervention. Nevertheless, for a nontrivial proportion of CRPs, the nature of the high-acuity work may lead to a range of adverse outcomes. Distress reactions that may arise from working in acute care settings span moral distress [2], burnout, attrition, poor work quality, and poor psychological functioning (cf. research in psychiatric settings [3]). Another potential outcome, though likely less common, is posttraumatic stress disorder (PTSD).

Research teams include individuals with varied training backgrounds and personal and professional experiences. CRPs with clinical backgrounds, such as nurses or physicians, likely have different levels of preparation for and familiarity with navigating potentially distressing and traumatic situations inherent in acute care medical settings. While these experiences may buffer their psychological response to some extent, clinical training itself does not confer immunity to psychological distress. Even seasoned clinicians in acute care settings can and do experience adverse psychological health challenges in these high-stakes environments [4], and this can occur in the context of clinical research as well, where distress may be experienced not only firsthand but also vicariously (e.g., through the eyes and emotions of non-clinical colleagues). In addition, CRPs may be involved in long-term follow-up with patients and families managing chronic or lifelong disabilities (e.g., traumatic brain injury, spinal cord injury, stroke, transplant), which may compound psychological burden beyond sudden-death contexts. Differences may also emerge between early-career CRPs with relatively limited exposures versus those with decades of cumulative exposures. Moreover, cultural perspectives shape how distressing events are perceived and processed, underscoring the need for culturally informed support strategies.

## Looking ahead

Ensuring that CRPs remain healthy ensures that people prosper and science advances. To this end, there is a need for a large-scale observational study of research team members who work in acute care settings to understand their psychological experiences as they relate to clinical research. It would also be useful to ascertain their views on and experiences with receiving preventative psychological interventions and other sources of organizational support. Parallel efforts should employ mixed-methods approaches – including structured and semi-structured qualitative interviews – to capture nuanced experiences across roles. As part of this effort, risk and protective correlates could be identified for various outcomes (e.g., moral distress, burnout, attrition, resilience, PTSD), as well as differential risk across strata, such as CRPs with versus without a clinical background.

Ultimately, this information could be used to develop, test, and deploy a brief educational curriculum designed specifically for CRPs embedded in acute care settings. This brief training, if demonstrated to be useful, could be deployed through clinical trial networks and other clinical research consortia or integrated into existing research training courses, such as the commonly used Collaborative Institutional Training Initiative (CITI) Program. Indeed, currently, the CITI Program has little-to-no focus on maintaining the psychological health of the individual taking the training: the CRPs. By bolstering CRP training to include modules focused on psychological health, common (and less common) distress reactions could potentially be averted.

## Summary

The clinical research enterprise necessarily depends on a team of individuals with unique life experiences, educational trajectories, skills, and comfort levels. These and other factors may impact their views of – and psychological response to – various facets of acute care clinical research. The field must recognize and address the psychological risks inherent in this work. Supporting the psychological health of CRPs should be seen as both an ethical imperative as well as a strategic initiative for the advancement of practice-changing research: healthy CRPs contribute to cohesive, resilient teams, leading to high-quality, efficient research operations and scientific advancements.
